# Reply to ‘Machine learning detection of obstructive hypertrophic cardiomyopathy using a wearable biosensor’

**DOI:** 10.1038/s41746-019-0187-9

**Published:** 2019-12-10

**Authors:** Reinier van Mourik, Onur Dur, Stephen B. Heitner, Charles Wolfus, Eric M. Green, Marc J. Semigran

**Affiliations:** 1 Wavelet Health, Mountain View, CA USA; 2 Oregon Health Sciences University, Portland, OR USA; 3 MyoKardia, Inc., South San Francisco, CA USA

**Keywords:** Cardiac hypertrophy, Cardiomyopathies

**Replying to** R. Ueno et al. *npj Digital Medicine* 10.1038/s41746-019-0186-x (2019)

We thank Dr. Ueno for the interest in our article. In reply to his statistical concerns about the execution of the Leave-One-Group-Out cross-validation (LOGO CV) and its interpretation, we believe that the brevity of our explanation of the nested cross-validation procedure may have caused confusion, and we offer the following clarification regarding the number of CV folds and the inclusion of all 82 patients in the confusion matrix.

We evaluated our classifier using nested cross-validation,^1^ where the hyperparameters in each of the 82 LOGO CV folds were selected by 68-fold random patient split CV (68 folds were used to balance statistical precision and hardware efficiency). The LOGO CV method refers to the exclusion of a group of recordings from one patient. That is, for each of the 82 patients, we trained the classifier on the other 81 patients’ recordings and evaluated it subsequently on the excluded patient’s recordings; thus, the LOGO CV ran 82 folds. To ensure that the excluded patient is entirely new to the classifier, hyperparameter tuning must also exclude the patient. Thus, in each LOGO fold, hyperparameter tuning by cross-validation is performed on the 81-patient training set. This *nested* CV performs 68 folds of splitting 81 patients randomly into training (70%) and testing (30%) cohorts solely to select LOGO-fold-specific hyperparameters. These are then used during training of the classifier on the 81 patients, which is in turn used to classify the excluded patient’s recordings.

Since all of a patient’s recordings are evaluated by an instance of the classifier that has never seen the patient, all recordings of all patients can be included in the final confusion matrix.

By using nested cross-validation we have striven to prevent overestimating the model’s performance. A larger study, as Dr. Ueno suggests, can further validate these results.

To correct the results reported on Page 2, we would like to note that out of 64 volunteers, the automated beat detection quality control algorithm identified the PPG recordings in one subject to be technically inadequate. Therefore, the model correctly classified 18/19 patients with oHCM and 62/63 healthy volunteers (80/82 subjects total, 98% accuracy) as illustrated in Fig. 1d.
